# Comparing the Properties of Cellulose Nitrates Synthesized from *Miscanthus × giganteus* Stems and from Commercial Microcrystalline Cellulose

**DOI:** 10.3390/polym18131653

**Published:** 2026-07-02

**Authors:** Vera V. Budaeva, Anna A. Korchagina, Yulia A. Gismatulina, Evgenia K. Gladysheva, Polina A. Gorbatova, Anastasia A. Zenkova, Vladimir N. Zolotukhin, Gennady V. Sakovich

**Affiliations:** 1 Bioconversion Laboratory, Institute for Problems of Chemical and Energetic Technologies, Siberian Branch of the Russian Academy of Sciences (IPCET SB RAS), Biysk 659322, Russia; julja.gismatulina@rambler.ru (Y.A.G.);; 2 Biysk Technological Institute, Polzunov Altai State Technical University, Biysk 659305, Russia

**Keywords:** *Miscanthus × giganteus*, cellulose, microcrystalline cellulose, nitration, cellulose nitrates, properties

## Abstract

This paper reports new results on the synthesis and comparative characterization of cellulose nitrates (CNs) derived from *Miscanthus × giganteus* stems and from commercial microcrystalline cellulose (MCC). *Miscanthus* CNs synthesized by nitration with mixed sulfuric–nitric acids containing 16–20% water exhibit new functional properties: a high solubility in organic solvents (100% in acetone and 97–99% in alcohol–ether solvent) and a high viscosity (17–51 mPa·s), with a nitrogen content of 10.54–12.08 wt%. CNs from *Miscanthus × giganteus* are similar in nitrogen content and solubility to those from MCC (11.54% and 99%) but have a significantly greater viscosity (3 mPa·s), which is an undoubted advantage and considerably expands their potential application range. The solubility test of CNs synthesized from both sources demonstrated that *Miscanthus* CNs have a better film-forming ability. SEM analysis revealed a great difference in fiber length, despite the same cylindrical shape and observed aggregation: 1.0–2.0 mm for CNs from *Miscanthus* versus 40–60 μm for CNs from MCC. IR spectra of CNs from both sources showed the appearance of five new characteristic frequencies (1632–1633, 1273–1274, 823–826, 748, 677–686 cm^–1^ for *Miscanthus* CNs and 1659, 1277, 832, 747, 691 cm^–1^ for CNs from MCC), allowing the obtained compounds to be identified as nitric acid esters of cellulose. According to TGA/DTA analysis, the synthesized polymers have similarly high values of the onset temperature of both intense decomposition (197–198 °C) and narrow exothermic peaks (209–211 °C and 212 °C), respectively, indicating their high thermal stability. The combination of high solubility, viscosity, thermal stability and chemical purity of CNs derived from *Miscanthus × giganteus* stems suggests that strong thin films can be obtained and recommended for use in the manufacture of nitrocellulose membranes.

## 1. Introduction

Cellulose nitrates (CNs) are among the most important inorganic cellulose esters with a rich two-century history [1,2,3,4]. CNs are mainly used for industrial purposes [3,5]; however, in recent years, the rapid development of the economy and technologies has led to an active search for new areas of application, which requires the production of CNs with new functional properties. Particular attention is focused on the potential application of CNs in biomedicine [3,5]: the manufacture of thin-film membranes [6,7,8,9,10,11,12], including those for prenatal diagnosis and cancer cell detection [1], substrates for biosensors [13,14,15,16] and biosensors with improved analytical characteristics [17], as well as the fabrication of nitrocellulose membranes for microarrays [18,19], wound dressings [5,20], periodontal gels [21], electrophoresis films, and nonionic cosmetic film formers [1].

CNs with a nitrogen content of 11.5–12.2 wt% can be used in biomedicine due to a set of specific characteristics [3,6,7,15]. The ability of CNs to immobilize proteins is a pivotal property [1,6,9,10,12]. The high solubility in organic solvents [4,5,7,8,9,10], flexibility, and good film-forming ability [4,5,20] ensure the manufacturability, while biodegradability and low toxicity [12,21,22] determine the biocompatibility of the material.

The solubility of CNs in organic solvents, such as methyl acetate, acetone [5,7,8,9], N-methylpyrrolidone [21], N,N-dimethylacetamide, and methanol [12], is a key requirement for their biomedical application.

The expansion of CN applications is currently constrained by limited traditional raw materials (cotton [3,4,23,24,25,26,27] and wood [1,3,23,24,27]), as well as by the complexity of the production process for microcrystalline cellulose (MCC) [28,29,30], which necessitates an active search for new raw material resources [16]. Therefore, research on the synthesis of CNs from alternative, easily renewable raw materials [26,31,32,33,34,35,36], including MCC [31,33,34,37,38], becomes particularly relevant. A literature analysis of alternative plant raw materials for producing CNs shows no specific examples of the fabrication of membranes from nitrates of alternative celluloses [16], including *Miscanthus × giganteus* (MG).

We previously obtained CNs with a nitrogen content of 11.18–11.26% and a 94–95% solubility in an alcohol–ether mixture via the nitration of *Miscanthus × giganteus* cellulose (MGC) with a degree of polymerization (DP) of 1200–1350 using mixed sulfuric–nitric acids with an initial water content of 14% [39,40]. With a higher DP of 1770 and supplementary enzymatic hydrolysis of MGC, CNs were synthesized with a nitrogen content of 11.35–12.20% and a solubility of 41–94% [41]. However, the biomedical application requires a stably high solubility of CNs.

The present study aimed to investigate the nitration of high-viscosity MGC and commercial microcrystalline cotton cellulose (MCC) to obtain CNs with high solubility in organic solvents, as well as to study the characteristics and structure of highly soluble CNs from MGC versus CNs from MCC using modern methods.

## 2. Materials and Methods

### 2.1. Materials

The substrates used for the study were: cellulose isolated from the stems of nine-year-old *Miscanthus × giganteus* (grown in Penza Oblast, Russia) by a modified nitric acid method [41,42] and commercial MCC (Accent Microcell Private Limited, Gujarat, India).

#### Composition and DP of Cellulose Samples

The composition of cellulose samples (mass contents of α-cellulose, acid-insoluble lignin (Klason lignin), ash, and pentosans) and their DP were determined by standard chemical and physicochemical methods of cellulose analysis. The α-cellulose content was measured by treatment of cellulose samples with a 17.5% NaOH solution (CAS 1310-73-2, ≥99.0%, Scharlab, Sentmenat, Spain) and by quantification of the insoluble residue after washing with a 9.5% NaOH solution and water, followed by drying according to TAPPI Standard T 203 cm-22 [43]. The content of acid-insoluble lignin was determined by quantitative isolation of lignin through sequential hydrolysis of cellulose samples with concentrated mineral acids (hydrochloric acid, CAS 7647-01-0, ≥39.0%, OOO Khimleader, Barnaul, Russia; sulfuric acid, CAS 7664-93-9, ≥99.0%, OOO Khimleader, Barnaul, Russia) in accordance with TAPPI Standard T 222 om-83 [44]. The ash content was determined after incineration of cellulose samples in a Nabertherm L 3/11 muffle furnace with a B180 temperature controller (Nabertherm GmbH, Lilienthal, Germany) according to TAPPI Standard T 211 om-02 [45]. The content of pentosans was measured by treating cellulose samples with a 13% HCl solution with heating. The resultant furfural was collected in the distillate and its content was determined on a UNICO UV-2804 spectrophotometer (Princeton, NJ, USA), calibrated against xylose (CAS 58-86-6, ≥98.5%, OOO Khimleader, Barnaul, Russia) (a wavelength of 630 nm) with an orcinol–ferric chloride reagent (orcinol, CAS 504-15-4, ≥99%, OOO Khimleader, Barnaul, Russia; anhydrous iron(III) chloride, CAS 7705-08-0, ≥98.0%, OOO Khimreactivsnab, Ufa, Russia) [46]. The degree of polymerization (DP) was determined by measuring the efflux time of a cellulose solution in cadoxene (cadmium oxide, CAS 1306-19-0, ≥99.0%, OOO Khimreactivsnab, Ufa, Russia; ethylenediamine, CAS 107-15-3, ≥99.0%, AO LenReaktiv, Saint-Petersburg, Russia) using a VPZh-3 viscometer (ECROSKHIM Co., Ltd., Moscow, Russia) with a capillary diameter of 0.92 mm, as reported in [47].

Prior to nitration, the cellulose samples were dried in a Binder ED 23 drying chamber (Binder, Tuttlingen, Germany) at 60 ± 5 °C to a residual moisture content of no more than 5%. The moisture content of the cellulose samples was measured using an Ohaus MB23 moisture analyzer (Pine Brook, NJ, USA).

### 2.2. Nitration of Cellulose and Stabilization of CN Samples

**Figure 1:**
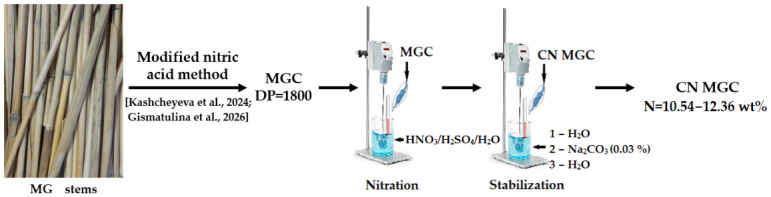
A schematic of the synthesis of CNs from *Miscanthus × giganteus* stems. Modified nitric acid method [41,42].

Dry MGC samples weighing 7 g were treated with mixed sulfuric–nitric acids. Nitration was carried out by varying the initial mass content of water in the mixed acid from 14% to 20% at 25–30 °C for 40 min, with the mass ratio of MGC to mixed acid being 1:50 [35,36,39,40,41]. Nitration of the commercial MCC sample was carried out with an initial water content of 14 wt% in the mixed acid and with an MCC-to-mixed acid mass ratio of 1:25, all other conditions being equal [35,36,39,40,41]. Nitration was carried out with continuous stirring using a HS-50A-Set overhead stirrer (Witeg Labortechnik GmbH, Wertheim, Germany). The obtained CN samples were filtered from the reaction mixture on a Büchner funnel and thoroughly washed with a large amount of distilled water at a temperature not exceeding 15 °C until the wash water gave a neutral reaction by the litmus test. In order to remove potential sulfate esters, the CN samples were stabilized by sequential treatment in water for 1 h, in a 0.03% sodium bicarbonate solution (CAS 144-55-8, ≥99.7%, OOO Khimleader, Barnaul, Russia) for 3 h, and again in water for 1 h until the filtrate reached neutrality according to the litmus test. All stabilization stages were performed at 90–95 °C with continuous stirring. Before analysis, the CNs were air-dried first for 24 h at room temperature and then dried in a drying oven for 1 h at 100 ± 5 °C; afterward they were weighed on an analytical balance (Explorer Pro EP214C, Ohaus, Langacher, Switzerland) to determine the actual yield [48] and then analyzed. The experiments are visualized in Figure 1.

It should be noted that CNs with a nitrogen content of less than 12.6 wt% are flammable solids (UN Class 4.1) [4]. Therefore, the synthesis of CNs requires precise control of the reaction parameters to ensure safe handling. The production process includes carefully balanced stoichiometric ratios of nitrating acids, controlled reaction conditions, and systematic purification procedures, which critically affect the purity and stability of the final product. To ensure safety, CNs must contain a certain amount of ethanol or water during storage. CNs should be stored in hermetically sealed containers, in a cool, dry, dark, and ventilated storage room at a temperature not above 30 °C, away from fire and heat sources.

### 2.3. Calculation of Actual Yield of CNs

The actual yield of CN samples (%) was calculated by Equation (1) [48]:

(1) $$\;W=\frac{m_{CN}}{m}\cdot 100$$

where *W* is the actual yield of CNs, %; *m_CN_* is the weight of the resultant CN sample, g; and *m* is the weight of the initial cellulose sample for nitration, g.

### 2.4. Physicochemical Analysis of CN Samples

CNs were analyzed by standard procedures. The mass content of nitrogen was determined by a quantitative method using iron(II) sulfate [2,27,29,31,35,36,39,40,41]. This method is based on the saponification of CNs (0.12 g) with concentrated sulfuric acid (25 mL) and the subsequent reduction of the resulting nitric acid by iron(II) sulfate (CAS 7783-85-9, OOO Khimleader, Barnaul, Russia) to nitric oxide (NO). The nitric oxide then reacts with an excess of iron(II) sulfate to form the [Fe(NO)]SO_4_ complex, which imparts a yellowish-pink color to the solution. This method is quite frequently used along with elemental analysis [26,28,30,32,37,38], and less frequently with the Kjeldahl method [27] and the Lunge method [33,34]. The standard deviation of 0.05% for the ferrous sulfate method is quite acceptable to evaluate the nitrogen mass content in the range of 10.50–12.50%. The degree of substitution (DS) was calculated according to [33,37]. The solubility of CNs (1 g) in acetone (50 mL) (CAS 67-64-1, ≥99.0%, OOO Khimleader, Barnaul, Russia) was determined by filtering the CN residue undissolved in acetone, followed by drying and weighing on an analytical balance [29,35,39,40,41]. The viscosity of CNs was determined by measuring the efflux time of a 2% solution of CN (1 g) in acetone (46 mL acetone and 4 mL H_2_O) from a VPZh-1 glass capillary viscometer (ECROSKHIM Co., Ltd., Moscow, Russia) [29,32,35,36,40,41]. The solubility in an alcohol–ether solvent was determined by dissolving CNs (1 g) in an alcohol–ether solvent (150 mL) at a 1:2 volume ratio of ethyl alcohol (CAS 64-17-5, ≥96.0%, OOO Khimleader, Barnaul, Russia) to diethyl ether (CAS 60-29-7, ≥99.0%, OOO Khimleader, Barnaul, Russia), followed by filtration, drying, and weighing of the undissolved residue on an analytical balance [29,31,35,36,40,41].

The experimental results for the determination of the composition and degree of polymerization (DP) of the cellulose samples, as well as for the nitration, stabilization, and physicochemical properties of the CN samples, were obtained in triplicate, statistically processed using standard methods with Microsoft Office Excel 2019, and are significant.

### 2.5. Structural Characterization of Cellulose and CN Samples

#### 2.5.1. SEM of Cellulose and CN Samples

Scanning electron microscopy (SEM) was used to examine the surface morphology and determine the fiber dimensions of the cellulose and CN samples after sputtering Ag with a layer thickness of 1–5 nm. The study was conducted at magnifications of ×50 to ×10,000 [35,36,39,40,41]. Some studies were carried out in a similar manner in [2,22,24,26,29,31,32,33,34,38].

#### 2.5.2. FTIR Spectroscopy of Cellulose and CN Samples

FTIR spectroscopy was used to acquire information on the molecular structure of the cellulose and CN samples [2,24,25,26,28,29,30,31,32,33,34,35,36,37,38]. An Infralum FT-801 FTIR spectrometer (OOO NPF Lumex Sibir, Novosibirsk, Russia) was employed with single-pass attenuated total reflectance (ATR) in the frequency range of 4000–600 cm^−1^. The recorded spectra were normalized to the absorption peak at 1160 cm^−1^, corresponding to the C–O–C glycosidic stretch of cellulose. This peak is used for normalization because this group is not affected and not involved in the substitution reaction [26].

#### 2.5.3. Coupled Thermogravimetric and Differential Thermal Analysis (TGA/DTA) of Cellulose and CN Samples

The thermal behavior of the cellulose and CN samples was studied under the following conditions: a sample weight of 0.5 mg, a heating rate of 10 °C/min, a temperature range from 0 °C to 600 °C (maximum temperature 350 °C), and nitrogen as inert atmosphere [35,36,40,41]. The thermal behavior of the studied samples was investigated in a similar manner in [25,28,29,31,32,33,34,37,38].

## 3. Results and Discussion

### 3.1. Physicochemical Properties of Cellulose Samples

**Figure 2:**
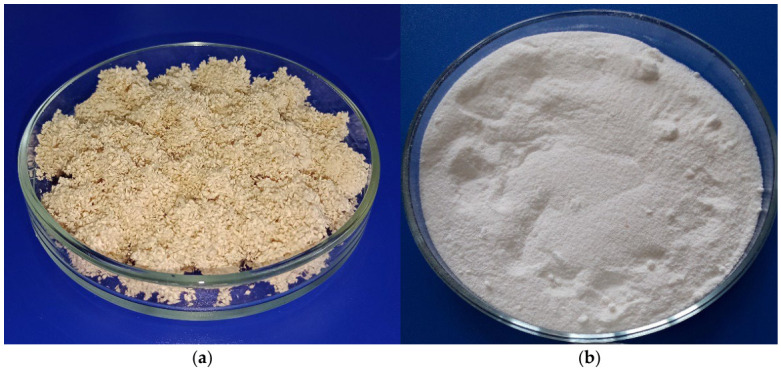
Appearance of (**a**) MGC and (**b**) commercial MCC.

Visually, the MGC sample (Figure 2a) appears as a light-brown short-fiber mass without foreign inclusions, in contrast to the commercial MCC sample representing a homogeneous white powder (Figure 2b).

Table 1 presents the composition of the MGC and commercial MCC samples in comparison with literature data on cellulose from avocado seeds [31].

According to Table 1, the MGC and commercial MCC samples differ significantly in DP (1800 vs. 240), with close values for the α-cellulose content of 93.0% and 96.3%, respectively. The contents of non-cellulosics in the MGC sample (acid-insoluble lignin, pentosans, and ash) are 0.74%, 3.64%, and 0.22%, respectively, and are higher than those for the commercial MCC sample (0.19%, 0.86%, and 0.08%), which is due to the well-known purity of commercial MCC. The comparison of the compositions of the MGC cellulose and commercial MCC samples shows the advantage of the former.

In 2025, Aljafree et al. [22] discussed the positive effect of the mass content of residual lignin in CN precursors. According to [22], the residual lignin preserves the porous structure of cellulose and facilitates better diffusion of nitrating agents, acting as a protective layer and preventing excessive degradation of cellulose when nitrated. However, the problem of nitrated lignin removal should be solved at the CN stabilization stage because the decomposition temperature of nitrated lignin is lower than that of CN. Our previously obtained successful results on the synthesis of CNs from cellulose of *Miscanthus sacchariflorus* (Maxim.) var. Soranovskii [35,36] with a residual lignin content of 0.15–1.36 wt% confirm no negative effect of residual lignin on the thermal stability of CNs.

Due to the higher DP values, MGC encompasses a wider range of applications compared to commercial MCC. The DP of cellulose is known to determine cellulose application areas. In filter production, high DP values of cellulose or its esters provide the necessary retention capacity of ultrafiltration membranes and demonstrate minimum protein adsorption, which is particularly useful in biopharmaceutical purification processes. Therefore, new membranes made of MGC-based CNs can become a more sustainable alternative material in membrane protein separation processes. It is necessary to take into account the direct relationship between the mechanical properties of films and the DP of cellulose or its esters; therefore, it is essential that the DP of the polymer ensures a high tensile strength and a long service life of the film.

The comparison of the properties of the MGC sample with the reported data for avocado seed-derived cellulose [31] showed that the MGC sample under study surpasses avocado seed-derived cellulose in α-cellulose (93.0% vs. 89.96%) and acid-insoluble lignin (0.74% vs. 1.25%), with an almost identical level of pentosans (3.64% vs. 3.86%). It is important to note that avocado seed-derived cellulose was subjected to a bleaching step, yet the pentosan level remained unchanged.

Thus, the MGC sample meets the requirements for cellulose intended for nitration, indicating that thermally stable CNs with a wide range of properties can be obtained.

### 3.2. Physicochemical Properties of CN Samples

Table 2 presents the main functional properties of CN samples obtained by nitration of MGC with mixed acid (the initial water content was varied from 14 wt% to 20 wt%) as compared with the properties of CNs from commercial MCC (initial water content of 14 wt% in the mixed acid). Literature data [29,30,31] are additionally provided.

Four CN samples were synthesized via nitration of MGC with mixed acid as the initial water content was varied from 14 wt% to 20 wt% (Table 2): CN MGC-14, CN MGC-16, CN MGC-18, and CN MGC-20. These samples exhibited the following main functional properties: a nitrogen content of 10.54–12.36 wt%, a viscosity of 17–233 mPa·s, 9–99% solubility in alcohol–ether solvent, and an actual yield of 111–134%. All CN samples demonstrated 100% solubility in acetone, confirming that they are nitric acid esters of cellulose [5,48].

It is known that the nitrogen content range of 10.8–12.3 wt% is considered enough for CNs to be used in the preparation of nitrocellulose membranes with high protein affinity [5,6]. Therefore, the solubility in mixed alcohol–ether is critical in this case as the criterion for solubility in other solvents. The use of mixed acid containing 14 wt% water turned out to be insufficient to obtain a highly soluble CN sample. In fact, the resultant CN sample had a nitrogen content of 12.36 wt%, a viscosity of 233 mPa·s, and an actual yield of 134%, yet its solubility was only 9%. However, as the initial water content was further raised from 16 wt% to 20 wt%, the solubility of CN samples in the alcohol–ether solvent was observed to reach 97–99%, along with a consistent decrease in viscosity from 51 mPa·s to 17 mPa·s and in actual yield from 127% to 111%. The obtained alcohol–ether solutions of the CN samples CN MGC-16, CN MGC-18, and CN MGC-20 were dried in a thin layer on a flat surface to form films. The obtained films were found to have a homogeneous texture and a smooth and even surface, as well as high transparency, proving their good film-forming ability [4,5,20]. The acetone solutions of the CN samples had high transparency as well, but the resultant films after drying were opaque and homogeneous, as similarly reported in [9]. The obtained results on the solubility in alcohol–ether solvent and acetone suggest that three CN samples derived from MGC can be used as an assay component for biomedical applications.

The comparison of the main functional properties of CN samples obtained by nitration of MGC with mixed acid containing water in the range of 16–20 wt% with those of the CN sample from commercial MCC (initial water content of 14 wt%) shows that they all have similar nitrogen contents, 10.54–12.08 wt% vs. 11.54 wt%, and solubilities in alcohol–ether solvent, 97–99% vs. 99%. It is evident that the CN samples from MGC clearly exceed the CN sample from commercial MCC in viscosity, 17–51 mPa·s vs. 3 mPa·s, which is an undoubted advantage and significantly expands their potential application range, particularly in biomedicine: the manufacture of thin-film membranes [6,7,8,9,10,11,12], including those for prenatal diagnosis and cancer cell detection [1], substrates for biosensors [13,14,15,16] and biosensors with improved analytical characteristics [17], as well as the fabrication of nitrocellulose membranes for microarrays [18,19], wound dressings [5,20], periodontal gels [21], electrophoresis films, and nonionic cosmetic film formers [1]. The high viscosity of CNs from MGC implies lower solution concentrations to achieve the desired flowability during film formation and the required porosity of dry films, as well as their high thermal stability that is directly dependent on viscosity.

The solubility test of the CN from commercial MCC with the same concentration as CNs from MGC in mixed alcohol–ether showed that the resultant films could not be removed from the surface, as they had zero strength. Nitration of commercial MCC leads to CN with a low viscosity and a low film-forming ability due to the low DP of the initial commercial MCC, although study [29] reported the synthesis of CN with a viscosity of 17 mPa·s from commercial MCC.

The comparison of the main functional properties of experimental CN MGC-16 and CN MGC-18 with those of CN from commercial MCC [29] (Table 2) demonstrates the superiority of the CN MGC samples in terms of solubility in alcohol–ether solvent (97–99% vs. 90%) and viscosity (38–51 mPa·s vs. 17 mPa·s), with a minor difference in the nitrogen content for the preparation of membranes (11.60–12.08% vs. 12.50%). According to Table 2, the CN MGC-20 sample exceeds the CN sample from commercial MCC in terms of solubility in alcohol–ether solvent (97% vs. 90%) while having the same viscosity (17 mPa·s), with a noticeable difference in the nitrogen content (10.54% vs. 12.50%) [29]. The comparison of experimental CN MGC-16, CN MGC-18 and CN MGC-20 with the CN from commercial MCC [30] (Table 2) shows their absolute advantage in terms of the nitrogen content (10.54–12.08% vs. 3.72% (as determined by elemental analysis)). Note that the CN MGC-18 sample and the CN from commercial MCC [30] were obtained at the same water content in the mixed acid (18%), yet CN MGC-18 had three times more nitrogen.

Experimental CN MGC-16 and CN MGC-18 exhibit higher values of solubility in alcohol–ether solvent (97–99% vs. 90–93%) and of actual yield (118–127% vs. 112–114%) compared with the CN from avocado seed-derived MCC [31] (Table 2), with a minor difference in the nitrogen content (11.60–12.08% vs. 12.23–12.26%). The CN MGC-20 sample has a higher solubility in alcohol–ether solvent (97% vs. 90%) compared with the CN from avocado seed-derived MCC [31] (Table 2), with a significant difference in the nitrogen content (10.54% vs. 12.23–12.26%) and with a similar actual yield (111% vs. 112–114%). Unfortunately, no viscosity data are provided in [31]. The higher viscosity values of the CN samples from MGC compared with the literature data for the CN from MCC once again emphasize their advantage and expand their application range.

Thus, CNs with new functional properties were synthesized by nitration of MGC with a high DP (1800) using a commercial mixed sulfuric–nitric acid containing 16–20 wt% water: a high solubility in organic solvents (100% in acetone and 97–99% in alcohol–ether solvent) along with a high viscosity (17–51 mPa·s), with a nitrogen content of 10.54–12.08 wt%.

The comparison of the main functional properties of the CN samples from MGC with those of the CN sample from commercial MCC shows that they have similar nitrogen content, 10.54–12.08 wt% vs. 11.54 wt%, and solubility in alcohol–ether solvent, 97–99% vs. 99%. The CN samples from MGC exceed the CN sample from commercial MCC in viscosity, 17–51 mPa·s vs. 3 mPa·s.

Thus, the obtained results confirm the potential of the new, highly soluble, high-viscosity CNs derived from MG cellulose for use as a component of biomedical products, in particular for the manufacture of rapid tests.

### 3.3. SEM Results for Cellulose and CN Samples

**Figure 3:**
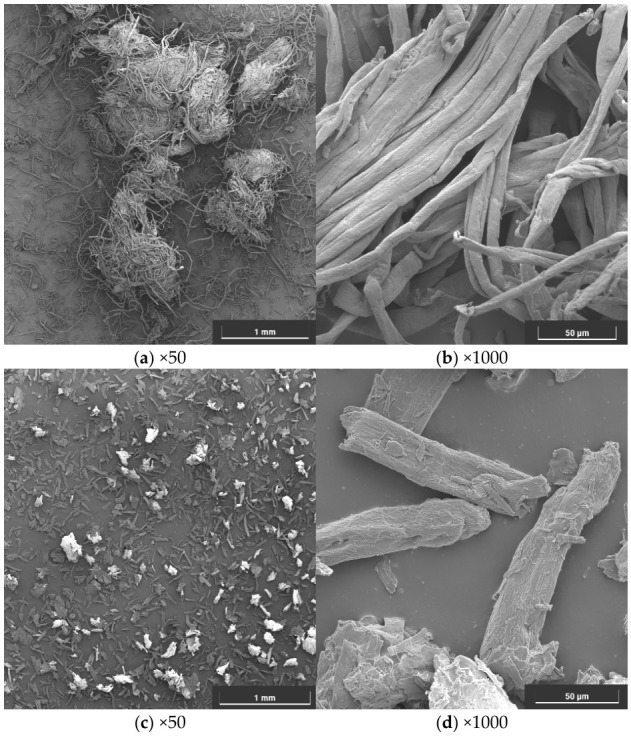
SEM images of (**a**,**b**) MG cellulose and (**c**,**d**) commercial MCC.

Figure 3 shows SEM images of the fibers of initial MGC samples and commercial MCC.

SEM examination of the initial MGC sample (Figure 3a,b) showed that the morphology of MGC is represented by different types of structural–dimensional elements—long tube-shaped fibers with “torn” edges, flat ribbon-like fibers, and single long and very wavy fibers, as well as fiber aggregates of different densities with a fiber thickness of 4 μm to 20 μm—as opposed to the commercial MCC sample (Figure 3c,d), chiefly consisting of short, individual, flat fibers 21–29 μm thick and their aggregates, similarly to the commercial MCC fibers reported in [29]. The aggregation of the MGC and commercial MCC fibers is possibly related to the residual lignin and pentosans present in the celluloses [25]. The surface of the MGC and commercial MCC fibers is predominantly smooth. Slight fibrillation along the length can be observed on the individual MGC fibers, manifesting as small areas with cracks.

**Figure 4:**
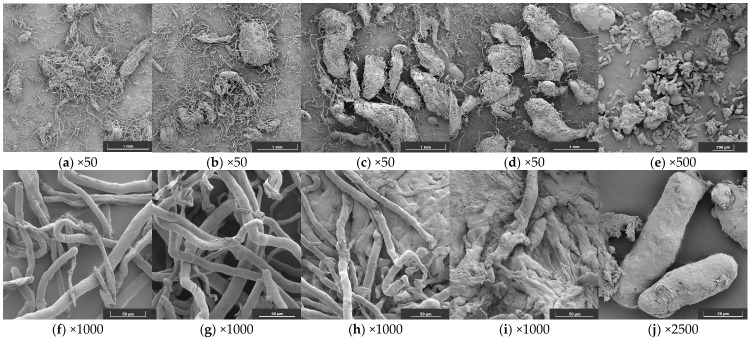
SEM images of CN samples obtained via nitration of MGC with mixed acid by varying initial water content: (**a**,**f**) 14%, (**b**,**g**) 16%, (**c**,**h**) 18%, (**d**,**i**) 20%. (**e**,**j**) CN from commercial MCC (14% water in mixed acid).

Figure 4 displays SEM images of the fibers of the synthesized CNs.

SEM examination of the CN samples synthesized from MGC (Figure 4a–d,f–i) showed that the long CN fibers chiefly acquire a cylindrical shape with a thickness of 3–19 μm after exposure of the initial MGC fibers to the mixed acid. Furthermore, aggregation of the cellulose nitrate fibers occurs, in which case the aggregation becomes more pronounced as the initial water content is raised from 14 wt% to 20 wt% in the mixed acid: the number and density of the conglomerates increase. Similarly to the CN fibers from MGC, the CN fibers from commercial MCC acquire a cylindrical shape with rounded ends after nitration (Figure 4e,j) but with a slight decrease in thickness to 18–20 μm. Multiple fiber aggregates appear in the overall mixture. The morphological surface of the CNs from both sources exhibits cracks and roughness caused by untwisting and swelling of the initial cellulose fibers during nitration [31,32,33,34]. Similar behavior and changes in the surface of cellulose fibers during the nitration process are also observed during the nitration of alternative feedstocks as compared to MCC [26,30,31,32,33,34,35,36,38]. A large difference in the fiber length is noteworthy: 1.0–2.0 mm for the CN from MGC (Figure 4e) versus 40–60 μm for the CN from commercial MCC (Figure 4j). The comparison with the literature data shows that the experimental CNs from commercial MCC are very similar in appearance to the CN fibers from commercial MCC [30], brown algae MCC [33], and esparto MCC [34].

### 3.4. IR Spectroscopy Results for Cellulose and CN Samples

**Figure 5:**
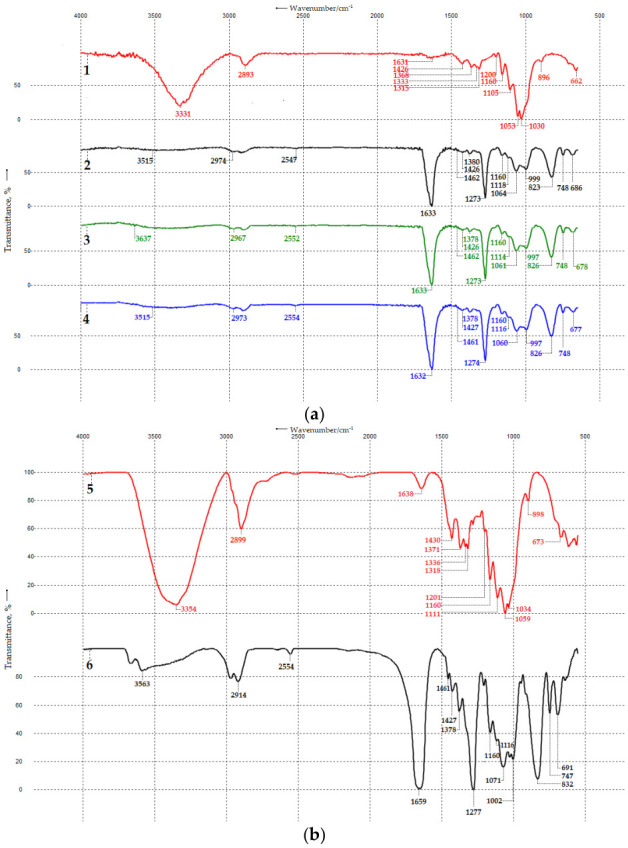
IR spectra of (**a**) (1) MGC and CN samples obtained through nitration of MGC with mixed acid by varying initial water content in mixed acid: (2) 16 wt%, (3) 18 wt%, and (4) 20 wt%; (**b**) (5) commercial MCC and (6) CN from commercial MCC (14% water content in mixed acid).

Figure 5 shows the IR spectra of the cellulose and CN samples. All obtained spectra were normalized to the absorption peak at 1160 cm^−1^, corresponding to the C–O–C glycosidic stretch of cellulose. This peak is used for normalization because this group is not affected and not involved in the substitution reaction [26].

The IR spectra of the initial cellulose samples (Figure 5a,b) illustrate the similarity of the characteristic frequencies of MGC (3331, 2893, 1631, 1426, 1160, 1053 cm^−1^) and commercial MCC (3354, 2899, 1638, 1430, 1160, 1059 cm^−1^), which correspond to O–H stretching, asymmetric and symmetric C–H stretching, O–H bending of adsorbed water, asymmetric CH_2_ bending, C–O–C stretching, C–O skeletal stretching, and β-glycosidic bond vibration of cellulose, respectively [27,28,30,31,32,33,34]. This confirms the identical chemical structure of the MGC samples and commercial MCC. The absence of additional pronounced absorption bands around 1735 cm^−1^ (carbonyl group stretching of hemicelluloses) and around 1512 cm^−1^ (aromatic ring vibration of lignin) [25,49,50] in the spectra of both cellulose samples indirectly corroborates the chemical analysis data. This indicates a high degree of purification and delignification efficiency of MGC, which is consistent with the low total content of non-cellulosics in MGC (4.6%).

The observed structural similarity of the MGC samples to commercial MCC is convincing instrumental evidence of the synthesis of high-quality MGC with the correct chemical structure, which is a fundamental basis for its competitiveness as a feedstock for chemical transformation, in particular into CNs. The acquired IR spectra of the MGC samples and commercial MCC are similar to those of commercial cotton cellulose [26,27,28,30,31], cotton stalk cellulose [25], and commercial MCC [29], and also show similarity to those of cellulose and MCC isolated from alternative plant raw materials [26,30,31,32,33,34,35,36,38].

The appearance of new absorption peaks in the IR spectra of the synthesized CNs (Figure 5a,b) unequivocally proves the chemical transformation of the initial MGC and commercial MCC samples [26,27,28,30,32]. The presence of the two most intense peaks around 1630 cm^−1^ (1632–1633 cm^−1^ for the CN from MGC and 1659 cm^−1^ for the CN from commercial MCC) and 1270 cm^−1^ (1273–1274 cm^−1^ for the CN from MGC and 1277 cm^−1^ for the CN from commercial MCC), associated with asymmetric (νₐsNO_2_) and symmetric (νsNO_2_) stretching vibrations, is unambiguous evidence of the formation of nitric acid esters. The broad intense peak around 820 cm^−1^ (823–826 cm^−1^ for the CN from MGC and 832 cm^−1^ for the CN from commercial MCC) corresponding to the N–O stretching vibration in NO_2_ further confirms the presence of the nitro group in the CN samples. The presence of less intense peaks around 740 cm^−1^ (748 cm^−1^ for the CN from MGC and 747 cm^−1^ for the CN from commercial MCC) and 680 cm^−1^ (677–686 cm^−1^ for the CN from MGC and 691 cm^−1^ for the CN from commercial MCC) corresponding to O–NO_2_ bending vibrations [26,28,30,32] indicates the high quality of the spectra and the purity of the synthesized polymers. The practical coincidence in the presence and position of all five peaks of the CN samples from MGC with the CN sample from commercial MCC shows the identity of the synthesized polymers at the molecular level. Note that the difference in the quality indicators of the experimental cellulose samples during nitration and subsequent stabilization did not lead to the appearance of extraneous functional groups or structural defects in the main polymer. The observed set of characteristic bands fully aligns with the spectral data for the CNs from commercial cotton cellulose [26,28,30,33,34] and commercial MCC [30], commercial CNs [24,32], and CNs from cellulose isolated from alternative plant raw materials and MCC [26,30,31,32,33,34,35,36,38].

The spectral changes after nitration (Figure 5) indicate the successful progression of esterification. The noticeable decrease in the intensity of the characteristic bands of the initial cellulose samples from both sources, the O–H stretching band (around 3330 cm^−1^) and the C–H stretching band (around 2900 cm^−1^), serves as a key sign. This decrease in intensity correlates with the degree of substitution of the hydroxyl groups by the nitro groups and serves as additional confirmation of the structural modification of the macromolecule [28,30,33,34].

### 3.5. TGA/DTA Results for Cellulose and CN Samples

**Figure 6:**
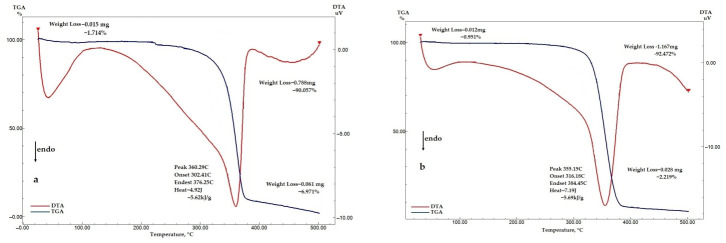

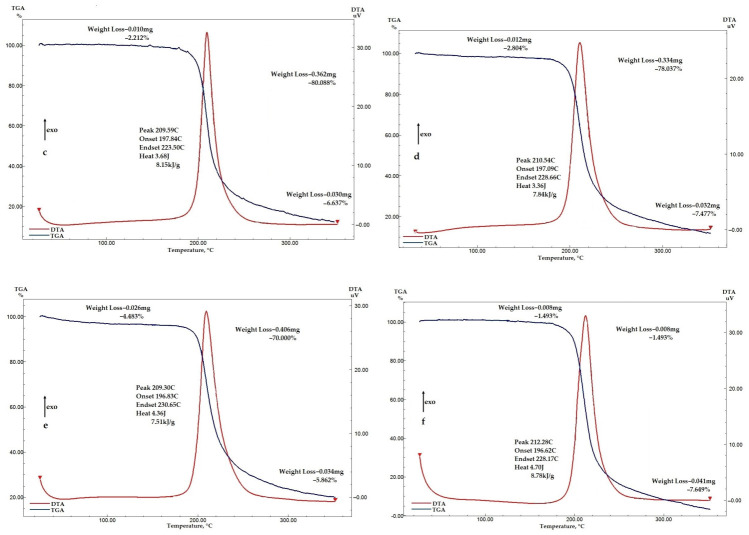
TGA curves of (**a**) MGC, (**b**) commercial MCC; CNs obtained via nitration of MGC with mixed acid by varying the initial water content in mixed acid: (**c**) 16 wt%, (**d**) 18 wt%, and (**e**) 20 wt%; and (**f**) CN from commercial MCC (14% water content).

Figure 6 depicts the results of coupled TGA/DTA analysis of the cellulose and CN samples.

Coupled TGA/DTA analysis (Figure 6) revealed a similar three-stage mechanism of thermal decomposition of the MGC samples (Figure 6a) and commercial MCC (Figure 6b). The initial stage (moisture desorption and retained water evaporation, as well as that of other volatiles [25,28,30]) occurs in the temperature range from the onset of the experiment to 140 °C for MGC and to 120 °C for commercial MCC, with a minimum weight loss of up to 1.7% and 0.9%, respectively. The key stage of cellulose pyrolysis ranges from 130 to 400 °C and corresponds to the deep thermal degradation of the crystalline structure of cellulose. In this range, the weight loss of both samples occurs up to 90% and 92%, respectively, due to depolymerization, dehydration, and cleavage of 1,4-glycosidic bonds, leading to the formation of a carbon residue [25,28,30]. Both cellulose samples have similar high onset temperatures of intense decomposition: 333 °C for MGC (Figure 6a) and 331 °C for commercial MCC (Figure 6b). The final stage of residue oxidation, corresponding to the temperature range of 400–500 °C for MGC and 410–500 °C for commercial MCC, is characterized by a minor weight loss of up to 6.9% and up to 2.2%, respectively. The endothermic peaks on the DTA curves (Figure 6a,b) at 360 °C for MGC and at 355 °C for commercial MCC, with a slight advantage for MGC, are accompanied by the same heat released value of 5.6 kJ/g. The obtained thermograms of both cellulose samples align with the typical decomposition curve of commercial cotton cellulose with an endothermic peak around 370 °C [28,30] and also exceed the endothermic value for cellulose from cotton stalk (347 °C [25]) and commercial MCC (330 °C [30]), which confirms their similarity and indicates the excellent thermal stability and purity of MGC and commercial MCC. The obtained results indicate that thermally stable CNs can be obtained from MGC over a wide range of properties.

TGA/DTA (Figure 6) revealed that, regardless of the initial water content in the mixed acid, the thermal decomposition of the CN samples obtained from MGC and commercial MCC proceeds through several stages [28,30]. The narrow exothermic decomposition peak of the CN samples from MGC occurs at 209–211 °C, and decomposition continues up to 280 °C, with the weight of the CN samples from MGC decreasing to 70–80% (Figure 6c–e). The narrow exothermic decomposition peak of the CN sample from commercial MCC occurs at 212 °C, and the decomposition continues up to 280 °C similarly to the CNs from MGC, with the weight of the CN sample from commercial MCC decreasing to 88% (Figure 6f). Further, the CN samples from both sources continue to decompose with a minor residue weight loss of 5.9–7.5% for the CNs from MGC and of 7.6% for the CN from commercial MCC.

The comparison of the synthesized CNs from both sources in terms of the onset temperature of intense decomposition with the CN from commercial cotton cellulose (197–198 °C vs. 193 °C [30]; 197–198 °C vs. 195 °C [32]; 197–198 °C vs. 195 °C [33]); with CN from commercial MCC (197–198 °C vs. 209 °C [30]; 197–198 °C vs. 188 °C [33]); with CNs from alternative sources, such as bitter bamboo stems (197–198 °C vs. 194 °C [32]), brown algae (197–198 °C vs. 197 °C [33]), and *Miscanthus sacchariflorus* (Maxim.) var. Soranovskii (197–198 °C vs. 201 °C [35] and 197 °C [36]); and the CNs from alternative MCC sources, such as brown algae (197–198 °C vs. 189 °C [33]), esparto (197–198 °C vs. 197 °C [34] and 190 °C [37]), giant reed (197–198 °C vs. 187 °C [37]), and palm leaves (197–198 °C vs. 187 °C [37]) shows the proximity of the obtained values, which in turn indicates the similarity of their thermal stability, high chemical purity, and energetic nature [3,30]. It was found that the CNs from MGC and the CN from commercial MCC have high specific heats of decomposition of 7.51–8.15 kJ/g and 8.78 kJ/g, respectively, which corroborates the energetic nature of the polymers [24]. The obtained data are in good agreement with the TGA/DTA data reported for CNs from alternative raw materials: 8.67 kJ/g for avocado seeds [31], about 7.0 kJ/g for CNs from avocado seed-derived MCC [31], and 9.24–11.04 kJ/g for *Miscanthus sacchariflorus* (Maxim.) var. Soranovskii [35,36]. The set of the characteristics of the degradation behavior and the high values of the specific heat of decomposition indicate the high chemical purity and thermal stability of the synthesized polymers [28,33,34]. When comparing with the results of other studies (all measurements were performed at the same heating rate), it should be borne in mind that small discrepancies may be due to differences in the TGA/DTA instrument used.

### 3.6. Potential Applications of CNs from MGC

The properties of the experimental CN samples from MGC versus the CN from commercial MCC are summarized in Table 3.

Due to the acceptable nitrogen content (11.60–12.08 wt%) and high solubility in organic solvents (97–99% in alcohol–ether solvent and 100% in acetone), along with the high viscosity (17–51 mPa·s) significantly exceeding that of the CN from commercial MCC (3 mPa·s), the synthesized CNs from MGC (Table 3) can be recommended for use in biomedicine for the manufacture of thin-film membranes [6,7,8,9,10,11,12], including those for prenatal diagnosis and cancer cell detection [1], substrates for biosensors [13,14,15,16] and biosensors with improved analytical characteristics [17], as well as in the fabrication of nitrocellulose membranes for microarrays [18,19], wound dressings [5,20], periodontal gels [21], electrophoresis films, and nonionic cosmetic film formers [1].

## 4. Conclusions

For the first time, cellulose nitrates (CNs) with new functional properties were synthesized in this work by nitration of *Miscanthus × giganteus* cellulose (MGC) with a high degree of polymerization (1800) using commercial mixed sulfuric–nitric acids with an initial water content of 16–20%. The resulting CNs exhibited high solubility in organic solvents (100% in acetone and 97–99% in alcohol–ether solvent) along with high viscosity (17–51 mPa·s), a nitrogen content of 10.54–12.08 wt%, and an actual yield of 111–127%.

Comparison of the main functional properties of CNs from MGC with those of CNs from commercial microcrystalline cellulose (MCC) revealed similar solubility in organic solvents (97–99% and 99% in alcohol–ether solvent, respectively, and 100% in acetone) and a similar nitrogen content (10.54–12.08% vs. 11.54%). However, CNs from MGC were significantly superior in viscosity (17–51 mPa·s vs. 3 mPa·s). The solubility test demonstrated that alcohol–ether solutions of MGC-derived CNs formed transparent films with a homogeneous texture and smooth surface, whereas MCC-derived CN films had zero strength and could not be detached from the casting surface, confirming the better film-forming ability of MGC-based CNs due to the high DP of the starting MGC.

SEM analysis revealed that CNs from both sources had a similar cylindrical shape with fiber aggregation but showed a large difference in fiber length: 1.0–2.0 mm for the CNs from MGC vs. 40–60 μm for the CN from MCC. IR spectroscopy confirmed the identity of the chemical structures of both celluloses and the appearance of five new characteristic peaks in the spectra of CNs, indicating the formation of nitric acid esters of cellulose. TGA/DTA analysis showed a high thermal stability of both types of CNs, with similar onset temperatures of intense decomposition (197–198 °C) and narrow exothermic peaks (209–211 °C and 212 °C, respectively). The weight loss of about 80% and high specific heats of decomposition (7.51–8.15 kJ/g for the CNs from MGC and 8.78 kJ/g for the CN from MCC) confirmed the high chemical purity and energetic nature of the synthesized polymers.

The combination of high solubility, viscosity, thermal stability, and chemical purity of cellulose nitrates derived from *Miscanthus × giganteus* stems suggests that strong thin films can be produced and recommended for use in the manufacture of nitrocellulose membranes.

## Figures and Tables

**Table 1:** The composition of MGC and commercial MCC samples in comparison with the reported data for cellulose from avocado seeds [31].

Sample Name	* Content, wt%				DP	Ref.
	α-Cellulose	Acid-Insoluble Lignin	Pentosans	Ash		
MGC	93.0 ± 0.5	0.74 ± 0.05	3.64 ± 0.05	0.22 ± 0.05	1800 ± 10	Present study
Commercial MCC	96.3 ± 0.5	0.19 ± 0.05	0.86 ± 0.05	0.08 ± 0.05	240 ± 10	Present study
Cellulose from avocado seeds	89.69 ± 1.05	1.25 ± 0.20	3.86 ± 0.34	no data	no data	[31]

Note: * on an oven-dry basis; MGC, cellulose from *Miscanthus × giganteus* stems; MCC, commercial microcrystalline cotton cellulose (Accent Microcell Private Limited, India); DP, degree of polymerization.

**Table 2:** Main functional properties of CN samples obtained by nitration of MGC with a varying initial water content of 14 wt% to 20 wt% in the mixed acid compared with those of CNs from commercial MCC (initial water content of 14 wt% in the mixed acid), as well as with the literature data on CNs from commercial MCC [29,30] and avocado seed-derived MCC [31].

Sample Name	Actual Yield, %	Characteristics				Ref.
		Nitrogen Content, wt%	Viscosity of 2% Solution in Acetone, mPa·s	Solubility in Acetone, %	Solubility in Alcohol–Ether Solvent, %	
CN MGC-14	134 ± 5	12.36 ± 0.05	233 ± 2	100 ± 0.5	9 ± 2	Present study
CN MGC-16	127 ± 5	12.08 ± 0.05	51 ± 2	100 ± 0.5	97 ± 2	
CN MGC-18	118 ± 5	11.60 ± 0.05	38 ± 2	100 ± 0.5	99 ± 2	
CN MGC-20	111 ± 5	10.54 ± 0.05	17 ± 2	100 ± 0.5	97 ± 2	
CN MCC	106 ± 5	11.54 ± 0.05	3 ± 2	100 ± 0.5	99 ± 2	
CN MCC (comm.)	no data	12.50 ± 0.3	17 ± 1.2	no data	90 ± 7	[29]
CN MCC (comm.)	no data	3.72	no data	no data	no data	[30]
CN from avocado seed-derived MCC	114	12.23 ± 0.94	no data	no data	90.36 ± 1.91	[31]
	112	12.26 ± 0.93	no data	no data	93.28 ± 2.34	

CN, cellulose nitrate; MGC, cellulose from *Miscanthus × giganteus* stems; MCC, commercial microcrystalline cotton cellulose (Accent Microcell Private Limited, India).

**Table 3:** Properties of the experimental CNs from MGC versus CN from commercial MCC.

Sample Name	Actual Yield, %	Nitrogen Content, wt%/DS	Viscosity of 2% Solution in Acetone, (mPa·s)	Solubility in Alcohol–Ether Solvent, %	Solubility in Acetone, %	Morphology	IR, cm^−1^	TGA/DTA
								T_Onset_/T_Peak_, °C/Q, kJ/g
CN MGC-16 (MGC DP of 1800)	127 ± 5	12.08 ± 0.05/2.29	51 ± 5	97 ± 2	100 ± 0.5	long (1.0–2.0 mm) cylinder-shaped fibers 3–19 μm thick; rough surface	1633, 1273, 823, 748, 686	198/210/8.15
CN MGC-18 (MGC DP of 1800)	118 ± 5	11.60 ± 0.05/2.14	38 ± 5	99 ± 2	100 ± 0.5		1633, 1273, 826, 748, 678	197/211/7.84
CN MGC-20 (MGC DP of 1800)	111 ± 5	11.54 ± 0.05/2.12	17 ± 5	97 ± 2	100 ± 0.5		1632, 1274, 826, 748, 677	197/209/7.51
CN MCC (MCC DP of 240)	106 ± 5	11.54 ± 0.05/2.12	3 ± 2	99 ± 2	100 ± 0.5	short (40–60 μm) cylinder-shaped fibers 18–20 μm thick; rough surface	1659, 1277, 832, 747, 691	197/212/8.78

Note: MGC, cellulose from *Miscanthus × giganteus* stems; MCC, commercial microcrystalline cotton cellulose (Accent Microcell Private Limited, India); CN, cellulose nitrate; DS, degree of substitution [48]; T_Onset_, onset temperature of intense decomposition of the sample, °C; T_Peak_, peak temperature of intense decomposition of the sample, °C; Q, specific heat of decomposition of the sample, kJ/g.

## Data Availability

The original contributions presented in this study are included in the article. Further inquiries can be directed to the corresponding author.
